# RISKY DECISION-MAKING AND ALZHEIMER’S DISEASE FAMILY MEDICAL HISTORY

**DOI:** 10.1093/geroni/igae098.3706

**Published:** 2024-12-31

**Authors:** Heaven Cauble, Sheila Black

**Affiliations:** University of Alabama, Tuscaloosa, Alabama, United States; University of Alabama, Tuscaloosa, Alabama, United States

## Abstract

Alzheimer’s Disease is associated with a decline in multiple cognitive domains including executive function (EF). This decline in EF often results in a decline in decision-making capacity (DMC). It can affect daily functioning in every aspect of life and oftentimes has extreme consequences related to healthcare and financial decisions. However, unlike other impairments of AD (e.g., memory and executive function), DMC has not been extensively researched as an early symptom. By studying people who are genetically at-risk of developing AD via family, we may be able to detect early differences in DMC as a possible result of genetic predisposition of AD. 60 middle-aged adults were recruited into groups based on family medical history of AD and completed the Game of Dice Task, a gambling task that measures risky decision-making. We found that family medical history of AD contributes to variances between scores, indicating that having an increased genetic predisposition to AD is associated with risky decision-making in mid-adulthood for those individuals. This research could support looking at decision-making capacity as a possible early symptom of AD, thus allowing for earlier intervention.

